# The complete mitochondrial genome of De Brazza’s monkey (*Cercopithecus neglectus*)

**DOI:** 10.1080/23802359.2021.1881929

**Published:** 2021-03-01

**Authors:** Yaohua Yuan, Guangyao Geng, Qunxiu Liu

**Affiliations:** Endangered Species Conservation and Research Centre, Shanghai Zoological Park, Shanghai, China

**Keywords:** *Cercopithecus neglectus*, mitochondrial genome, phylogenetic analysis

## Abstract

The complete mitochondrial genome of *Cercopithecus neglectus* was described. The mitogenome is 16,490 bp in length and consists 13 protein-coding genes (PCGs), 22 transfer-RNA genes, two ribosomal-RNA genes, and one non-coding region. All the 13 PCGs were 11,398 bp in length with most common start codon of ATG and termination codon of TAA. The overall GC content was 42.5%. The result of phylogenetic analysis showed that the relationship of *C. neglectus* was close to *C. mona*, *C. pogonias*, *C. wolfi* and *C. denti*.

## Background

De Brazza’smonkey (*Cercopithecus neglectus* Schlegel, 1876) was assessed to be Least Concern (LC) by IUCN (2016) and was listed in Appendix II of CITES. This species distributes widely in Angola, Cameroon, Equatorial Guinea, Gabon, Congo, Central African Republic, Uganda, Kenya, Sudan and Ethiopia (Mwenja et al. 2019). Deforestation and habitat loss are dominant factors causing the *C. neglectus* population to decline (Brennan 1985). The classification of the has been a major challenge for primate taxonomists (Groves 2001; Butynski 2002; Grubb et al. 2003),and the phylogenetic status of *C. neglectus* with other guenons species, should be concentrated. In this study, we sequenced the complete mitochondrial genome of *C. neglectus* and estimated the phylogenetic status of 17 *Cercopithecus* species. We hope to provide valuable information for the classification of *Cercopithecus* species (Ayoola et al. 2019).

## Materials and methods

The blood sample was collected from a female *C. neglectus* in Shanghai Zoological Park (longitude-121.36319, latitude-31.192682), Shanghai, China in March 2020. The sampling process was approved by the welfare and ethical committee in Shanghai Zoological Park. A specimen was deposited at the Endangered Species Conservation and Research Center of Shanghai Zoological Park (Liu Qunxiu, liuqunxiu2019@163.com) under the voucher number SH0008.

Total genomic DNA was extracted from the blood sample by Ezup blood kit (developed by Shanghai Sango Biotech Company, No.B518253). Based on closely related species, degenerate premiers were designed for PCR-amplification. The nucleotide sequences and relative PCR primers are shown in Table 1.

**Table 1.** Sequences of primers used in amplification of the complete mitochondrial genome in *C. neglects*.

Number	Primer sequences (5’-3’)	Gene region
1	1-F 5′-GCAAGACACTGAAGATGCCTAGA-3′ 1-R 5′-CGTGCTCGGGTGATTTTATT-3′	25–1236 = 1212
2	2-F 5′-GACCTACACTCGGAAGATCTCAATA-3′ 2-R 5′-TGCTTAGGCTTGTGCAGTCTTAG-3′	1047–2293 = 1247
3	3-F 5′-ATTGACCTGTCCGTGAAGAGA-3′ 3-R 5′-AGCTATGAAAAATAAGGCGAATG-3′	2092–3402 = 1311
4	4-F 5′-CGGCTCATTTAACCTTAACATACTC-3′ 4-R 5′-GTAATGGTTGGGTTATATGCTAGTG-3′	3195–4488 = 1294
5	5-F 5′-AAAACTAGCCCCCATCTCAATTA-3′ 5-R 5′-AGCCAGAAGCTTATGTTGTTTAGAC-3′	4288–5630 = 1343
6	6-F 5′-GTCAACCCGGTAGCTTATTAGG-3′ 6-R 5′-ATCACTGCTACTAACGAAATAAAGG-3′	5444–6716 = 1273
7	7-F 5′-CAAAGCTCACTTCATCGTTATATTC-3′ 7-R 5′-TAGCTGTGCAGTGCGCTTGTA-3′	6549–7721 = 1123
8	8-F 5′-AAACTACATTCACCGCCATACG-3′ 8-R 5′-AGATGTATTAGTAGGTGACCAGCTG-3′	7543–8454 = 912
9	9-F 5′-CATTTACACCTACCACCCAACTATC-3′ 9-R 5′-GTTGGGGTAATCAGAGTATAATGGT-3′	8208–9547 = 1340
10	10-F 5′-GCCGCTTGATACTGACACTTTG-3′ 10-R 5′-GGGATGAGAGTGGTTTCAAATAAG-3′	9328–10,552 = 1225
11	11-F 5′-CCAGCCAATATCACCTATACAATG-3′ 11-R 5′-CTAAGGCCAATGGATAGCTGTT-3′	10,405–11,710 = 1306
12	12-F 5′-CTCACCCCAATCATCCTTCTATC-3′ 12-R 5′-GTTGATGCCGATGGTGACTAT-3′	11,490–12,703 = 1214
13	13-F 5′-GGTGTTCCTACTCATTCGCTTTC-3′ 13-R 5′-TTTTTGGTTATACTACGGCGATG-3′ 13-FC 5′-AACCAACAATGCCCTACTACCTA-3′	12,511–13,878 = 1368 13,179
14	14-F 5′-CTCAAAAATTATCCAGCTCTCTATG-3′ 14-R 5′-TGCAAATAGGAAGTATCACTCTGGT-3′	13,697–14,978 = 1282
15	15-F 5′-AATCACTTTTCACCCCTACTACACA-3′ 15-R 5′-CGTGCGGACCAGAGATAAAAG-3′	14,798–15,900 = 1102
16	16-F 5′-AACTGTATCCGACATCTGGTTCTTA-3′ 16-R 5′-GTGTGGCTGTGCAAAGTGTT-3′	15,738–223

PCR was carried out under the following conditions: (1) 95 °C for 5 min (initial denaturation), (2) 94 °C for 30 sec (denaturation), (3) 58 °C for 30 sec (annealing), (4) 72 °C for 60 sec (extension), and then repeat (2)–(4) 38 cycles and a final extension at 72 °C for 10 min. Amplified PCR products were examined, purified and sequenced on ABI DNA sequencer (3730XL, America). Annotation was carried out by mitochondrial genome annotation (MITOS) (Bernt et al. 2013), tRNAscan-SE (Chan and Lowe 2019) and GeSeq – Annotation of Organellar Genomes web server (Tillich et al. 2017). Base composition was analyzed by MEGA 7.0 (Kumar et al. 2016).

The phylogenetic position of *C. neglectus* was assessed by comparison with the mtDNA sequences of different species from the NCBI database. Evolutionary phylogeny analysis was performed by MEGA 7.0 based on Maximum likelihood (ML) method because ML picks out the topology with the maximum likelihood based on good theoretical basis of statistics (Kumar et al. 2016). The phylogeny trees of 14 primates and 17 *Cercopithecus* species were set up by bootstrap of 500 iterations.

## Results

We obtained the complete mtDNA genome of an individual of *C. neglectus*. The mitogenome was submitted to NCBI GenBank and is available with accession number MW160353.

The complete mitogenome of *C. neglectus* is 16,490 bp in length. The base composition is A (31.6%), C (29.7%), T (25.8%) and G (12.8%) with 42.5% GC content. The mitogenome is consists of 37 genes [13 protein-coding genes (PCGs), two ribosomal RNAs (rRNA), 22 transfer RNAs (tRNA)]. All tRNAs have the typical cloverleaf structure.

All the 13 protein-coding genes (11,398 bp in length) were predicted by comparing with the previously published monkey mitochondrial genome sequences. We found similar gene arrangement and codon usage with other *Cercopithecidae* mitochondrial genome (Lei et al. 2010; Chang et al. 2016). A total of 10 of them use ATG as the start codon, while ND2, ND3 and ND5 use ATC, ATT and ATA separately. For the stop codon, there are ten protein-coding genes terminated with the typical stop condon of TAA, whereas ND1, ND2 and ND6 terminated with TAG, TAG and AGG (Table 2).

**Table 2.** The composition of the mitogenome of *C. neglectus*.

Gene	Position		Size	Codons		Anti-condon	Strand	Space/Overlap
	Start	End		Start	End			
tRNA-Phe	1	70	70			GAA	+	0
rrnS	135	377	949				+	0
tRNA-Val	1020	1088	69			TAC	+	0
rrnL	1089	2647	1559				+	0
tRNA-Leu	2648	2722	75			TAA	+	2
nad1	2725	3679	955	ATG	TAG		+	0
tRNA-Ile	3680	3748	69			GAT	+	−3
tRNA-Gln	3746	3817	72			TTG	−	1
tRNA-Met	3819	3886	68			CAT	+	0
nad2	3887	4928	1042	ATC	TAG		+	0
tRNA-Trp	4929	4994	66			TCA	+	7
tRNA-Ala	5002	5069	68			TGC	−	1
tRNA-Asn	5071	5143	73			GTT	−	32
Trna-Cys	5176	5242	67			GCA	−	−1
Trna-Tyr	5242	5307	66			GTA	−	12
cox1	5320	6888	1569	ATG	TAA		+	−28
Trna-Ser	6861	6929	69			TGA	−	3
Trna-Asp	6933	7000	68			GTC	+	1
cox2	7002	7685	684	ATG	TAA		+	23
Trna-Lys	7709	7774	66			TTT	+	2
atp8	7777	7977	201	ATG	TAA		+	−41
atp6	7937	8617	681	ATG	TAA		+	−1
cox3	8617	9400	784	ATG	TA(A)		+	0
Trna-Gly	9401	9468	68			TCC	+	0
nad3	9469	9812	344	ATT	T(AA)		+	2
Trna-Arg	9815	9879	65			TCG	+	0
nad4l	9880	10,176	297	ATG	TAA		+	−7
nad4	10,170	11,547	1378	ATG	T(AA)		+	0
Trna-His	11,548	11,616	69			GTG	+	0
Trna–Ser	11,617	11,675	59			GCT	+	0
Trna-Leu	11,676	11,746	71			TAG	+	6
nad5	11,753	13,555	1803	ATA	TAA		+	0
nad6	13,556	14,074	519	ATG	AGG		−	0
Trna-Glu	14,075	14,143	69			TTC	−	4
cob	14,148	15,288	1141	ATG	T(AA)		+	0
Trna-Thr	15,289	15,356	68			GTA	+	2
Trna-Pro	15,359	15,426	68			TGG	−	375
D-loop	15,802	15,981	180				+	509

**Figure 1.**
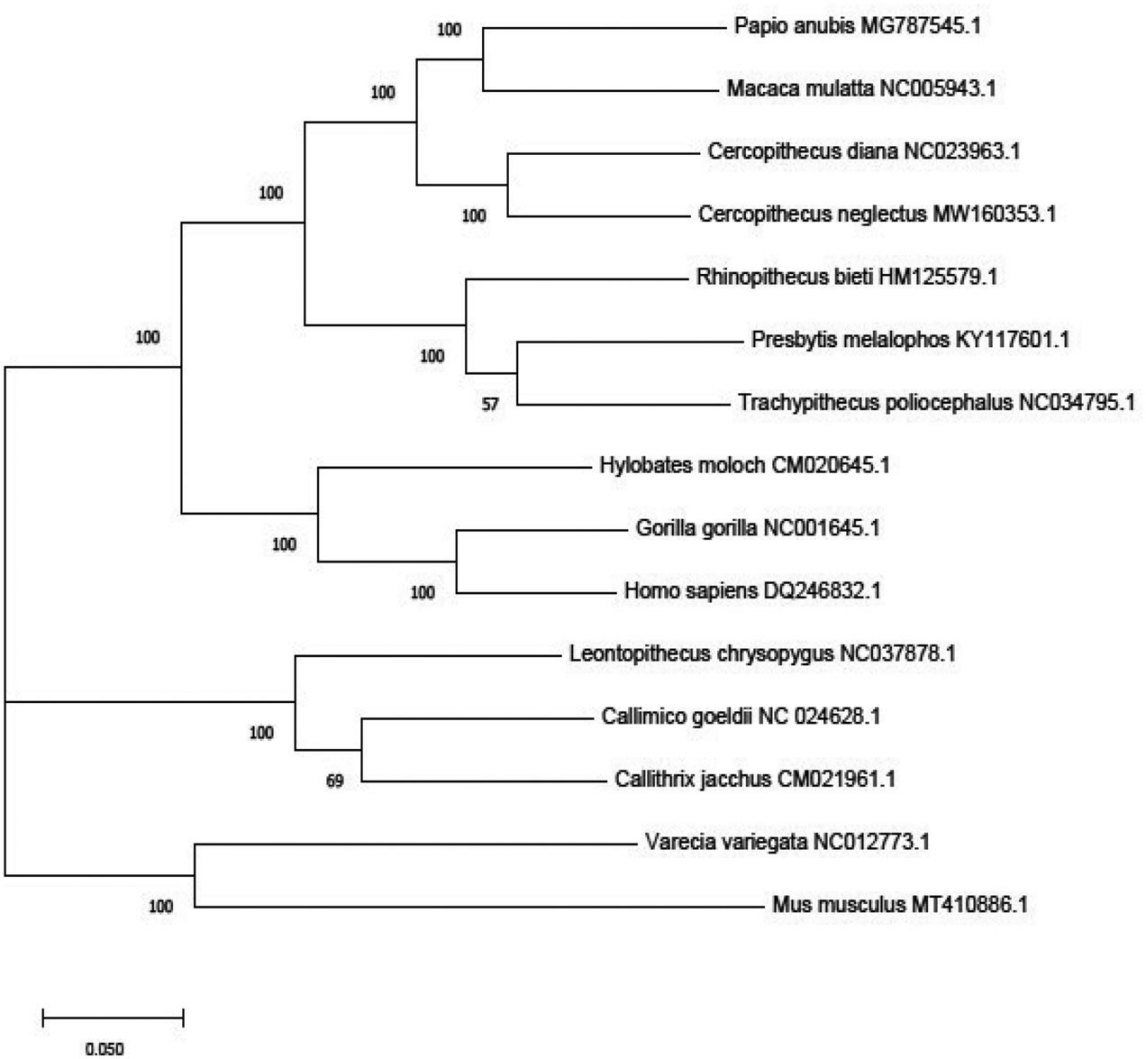
Phylogenetic relationship of 13 primate species based on Maximum likelihood model. Genbank accession Number: *Homo sapiens* (DQ246832.1), *Gorilla gorilla* (NC001645.1), *Hylobates moloch* (CM020645.1), *Varecia variegate variegate* (NC012773.1), *Presbytis melalophos mitrata* (KY117601.1), *Rhinopithecus bieti* (HM125579.1), *Papio anubis* (MG787545.1), *Macaca mulatta* (NC005943.1), *Cercopithecus diana* (NC023963.1), *Leontopithecus chrysopygus* (NC037878.1), *Callimico goeldii* (NC024628.1), *Callithrix jacchus* (CM021961.1), *Trachypithecus poliocephalus* (NC034795.1). *Mus musculus* (MT410886.1) was set as outgroup taxon.

**Figure 2.**
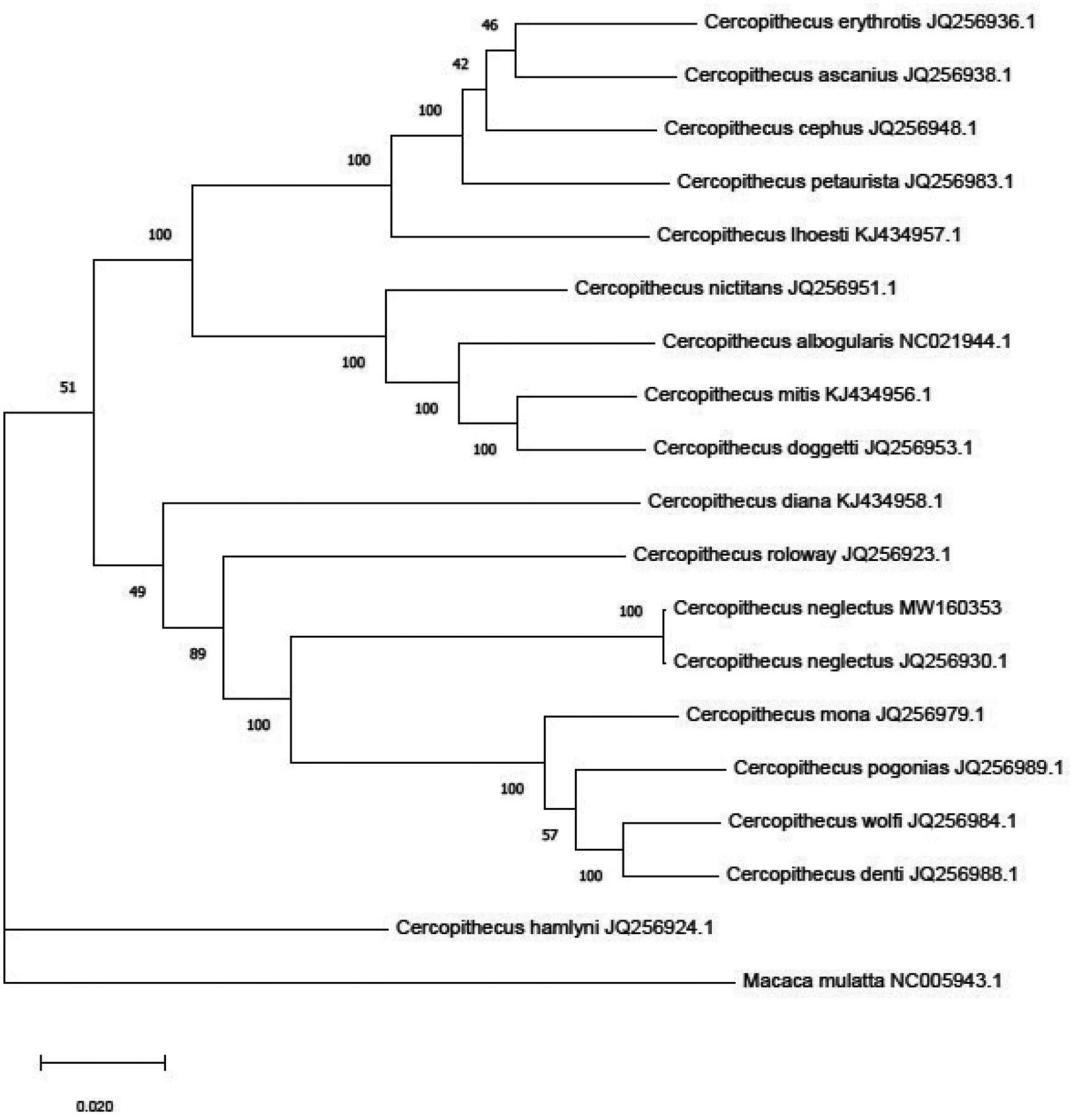
Phylogenetic relationship of 17 species of *Cercopithecus* based on Maximum likelihood model. Genbank accession number: *C. erythrotis* (JQ256936.1), *C. cephus* (JQ256948.1), *C. petaurista* (JQ256983.1), *C. ascanius* (JQ256938.1)*, C. lhoesti* (KJ434957.1), *C. nictitans* (JQ256951.1), *C. albogularis* (NC021944.1), *C. mitis* (KJ434956.1), *C. doggetti* (JQ256953.1), *C. hamlyni* (JQ256924.1), *C. diana* (KJ434958.1), *C.roloway* (JQ256923.1)*, C. neglectus* (MW160353)*, C. neglectus* (JQ256930.1), *C. mona* (JQ256979.1)*, C. pogonias* (JQ256989.1)*, C. wolfi* (JQ256984) *and C. denti* (JQ256988.1). *Macaca mulatta* (NC005943.1) was set as outgroup taxon.

For primates, *C. neglectus* was clustered together with *Papio Anubis* and *Macaca mulatta* with high bootstrap values (Figure 1). Among the *Cercopithecus* species, *C. neglectus* was close to *C. mona*, *C. pogonias*, *C. wolfi* and *C. denti* (Figure 2). In the phylogeny analyses of guenons species, Chatterjee et al. (2009) reported that *C. neglectus* forms a clade with *C. mona*, *C. hamlyni*, *C. solatus* and several Chlorocebus species. Disotell and Raaum (2004) found that *C. neglectus*, *C. pogonias* and *C. mona* were clustered together. Katerina et al. (2013) inferred the evolutionary relationships of all guenon taxa and reported that *C. neglectus* was close to *C. Diana*, *C. mona* and *C. pogonias wolfi* (Katerina et al. 2013). However, all the analyses on C. neglectus were based on subregion or partial mitochondrial genome sequence. In our study, we determined the first complete mitochondrial genome sequence of *C. neglectus* and estimated the phylogeny position of *C. neglectus* with other species. This study will help to better understand the features of *C. neglectus* mitogenome and provide more potential information for further evolutionary relationships within *Cercopithecus*.

## Data Availability

The genome sequence data that support the findings of this study are openly available in GenBank of NCBI at (https://www.ncbi.nlm.nih.gov/) under the accession no. MW160353 and available at: https://www.ncbi.nlm.nih.gov/nuccore/MW160353.1/.
